# Antioxidant and anti-inflammatory function of *Eupatorium adenophora* Spreng leaves (EASL) on human intestinal Caco-2 cells treated with tert-butyl hydroperoxide

**DOI:** 10.1038/s41598-024-61012-7

**Published:** 2024-05-07

**Authors:** Li Zheng-qiang, Ni Jun, Zhu Xin-yu, Zhang Chao-zhi, An Rui, Yang Xu, She Rong, Yang Xiao-yan

**Affiliations:** 1 Institute of Natural Antioxidants and Anti-Inflammation, Dali University, Dali, 671003 Yunnan China; 2 Institute of Eastern-Himalaya Biodiversity Research, Dali University, Dali, 671003 Yunnan China; 3 The Provincial Innovation Team of Biodiversity Conservation and Utility of the Three Parallel Rivers Region From Dali University, Dali, 671003 Yunnan China

**Keywords:** *Eupatorium adenophora* Spreng, Antioxidant, Anti-inflammatory agent, Caco-2 cell, Tert-butyl hydroperoxide (t-BHP), Cellular model of oxidative damage, LC–MS/MS, Biologics, Plant biotechnology

## Abstract

Chronic non-communicable diseases (CNCDs) pose a significant public health challenge. Addressing this issue, there has been a notable breakthrough in the prevention and mitigation of NCDs through the use of antioxidants and anti-inflammatory agents. In this study, we aim to explore the effectiveness of *Eupatorium adenophora* Spreng leaves (EASL) as an antioxidant and anti-inflammatory agent, and its potential applications. To construct a cellular model of oxidative damage and inflammation, Caco-2 cells were treated with tert-butyl hydroperoxide (t-BHP). The biocompatibility of EASL-AE with Caco-2 cells was assessed using the MTT assay, while compatibility was further verified by measuring LDH release and the protective effect against oxidative damage was also assessed using the MTT assay. Additionally, we measured intracellular oxidative stress indicators such as ROS and 8-OHdG, as well as inflammatory pathway signalling protein NFκB and inflammatory factors TNF-α and IL-1β using ELISA, to evaluate the antioxidant and anti-inflammatory capacity of EASL-AE. The scavenging capacity of EASL-AE against free radicals was determined through the DPPH Assay and ABTS Assay. Furthermore, we measured the total phenolic, total flavonoid, and total polysaccharide contents using common chemical methods. The chemical composition of EASL-AE was analyzed using the LC–MS/MS technique. Our findings demonstrate that EASL-AE is biocompatible with Caco-2 cells and non-toxic at experimental levels. Moreover, EASL-AE exhibits a significant protective effect on Caco-2 cells subjected to oxidative damage. The antioxidant effect of EASL-AE involves the scavenging of intracellular ROS, while its anti-inflammatory effect is achieved by down-regulation of the NFκB pathway. Which in turn reduces the release of inflammatory factors TNF-α and IL-1β. Through LC–MS/MS analysis, we identified 222 compounds in EASL-AE, among which gentianic acid, procaine and L-tyrosine were the compounds with high antioxidant capacity and may be the effective constituent for EASL-AE with antioxidant activity. These results suggest that EASL-AE is a natural and high-quality antioxidant and anti-inflammatory biomaterial that warrants further investigation. It holds great potential for applications in healthcare and other related fields.

## Introduction

Oxidative damage and inflammation are significant public health challenges worldwide: Oxidative stress is a biochemical process that occurs when the production of reactive oxygen species (ROS) exceeds a cell's ability to clear them^1,2^. This process leads to oxidative damage, which refers to the harm caused to biomolecules (such as proteins, lipids, and DNA) and cells due to the increased amount of ROS^3,4^. Additionally, ROS can activate the NFκB signaling pathway, triggering the synthesis and release of inflammatory factors, resulting in inflammation in cells and tissues^5^ Oxidative damage and inflammation are associated with various health issues, including cardiovascular diseases, cancers, neurodegenerative diseases, diabetes, and aging^6,7^, which one often classified as chronic non communicable diseases (CNCDs). These diseases pose significant public health challenges worldwide. To address this challenge, numerous scholars worldwide are actively researching and striving to discover high-quality natural and efficient antioxidant and anti-inflammatory substances. The present study is a contribution to this ongoing effort.

*Eupatorium adenophorum* Spreng (EAS): EAS is a perennial herb or semi-shrub of the Asteraceae family^8^. Originating from Mexico and Central America^9^, EAS has gained a reputation for its invasive nature, reproducing extensively and causing significant ecological damage worldwide^10^. Despite this, EAS continues to be utilized in traditional medicine across various regions, with reported applications in treating wounds, diabetes, inflammation, fever, jaundice, and dysentery^11–14^. Modern pharmacological studies have highlighted the diverse biological activities of EAS extracts, including antioxidant^15^, antibacterial^16^, anticancer^17^, wound healing^18^, analgesic^19^, antipyretic^20^, and anti-inflammatory activities^21^. However, regarding the mechanism of its antioxidant action, existing studies have focused on the assessment of free radical scavenging capacity in vitro and lack in vivo validation. In particular, there is a lack of assessment of antioxidant stress and anti-inflammatory activities and mechanisms at the cellular level, which is crucial for practical applications. Therefore, in order to draw precise and concise conclusions, this study was conducted using the aqueous extract from the leaves of EAS (EASL-AE), which, in preliminary findings, demonstrated the highest antioxidant activity among all extracts from the EAS category.

Oxidative stress cell model with Caco-2 induced by t-BHP: Human intestinal Caco-2 cells are commonly used as a model for intestinal epithelial cells due to their similarity to these cells, including microvilli and intercellular junctions. These cells have been extensively utilized in the research and development of natural antioxidant and anti-inflammatory substances^22–24^. Tert-butyl hydroperoxide (t-BHP) is a widely used organic peroxide, primarily used as an oxidant in organic synthesis. It is applied in various reactions such as oxidation, epoxidation, and decarboxylation. In the context of studying natural antioxidant and anti-inflammatory substances, t-BHP is frequently used to induce oxidative stress in Caco-2 cells, creating a model for oxidative stress^25–28^.

**Figure 1:**
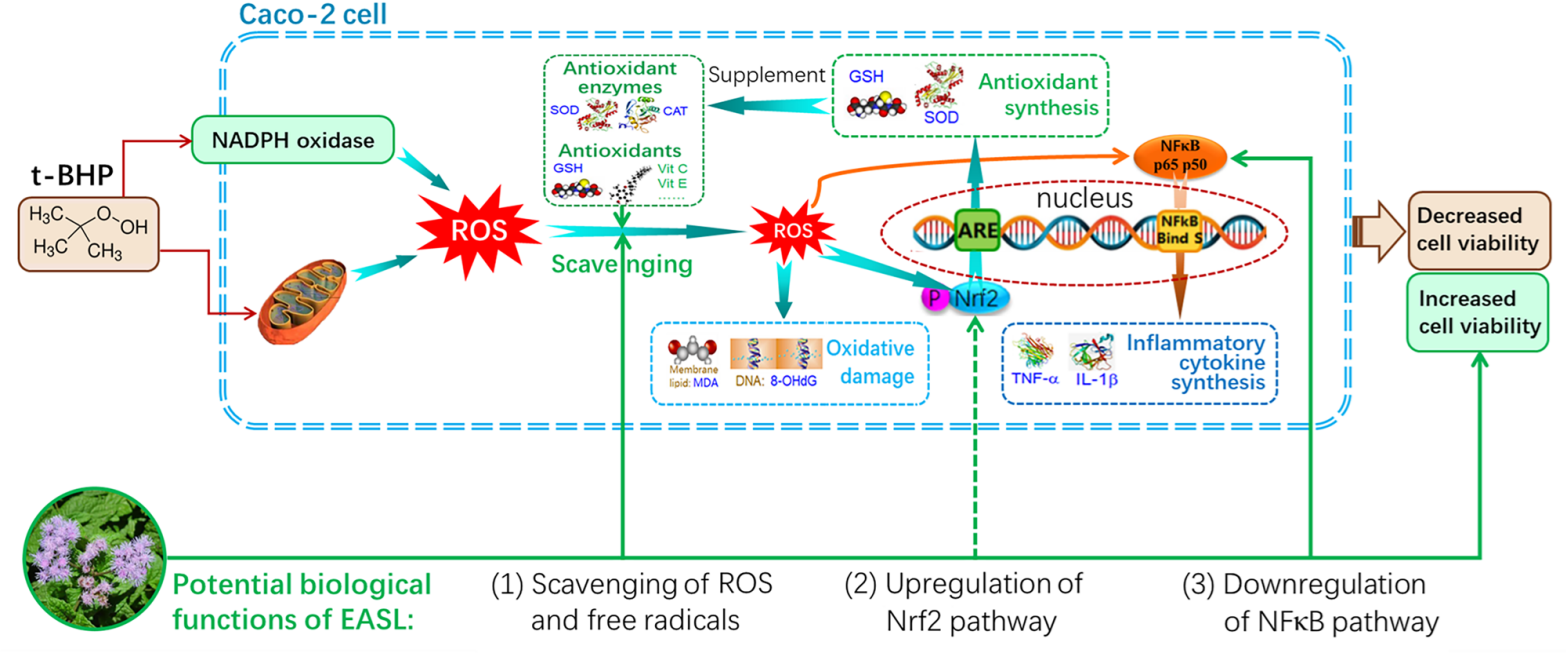
The scientific hypothesis of this study.

Our scientific hypothesis for this study: In order to conduct more scientific research, we have proposed the scientific hypothesis for this study, as shown in Fig. 1.

## Materials and methods

### Agents and instruments

All chemicals used were of high purity or suitable for analytical reagents. The reagents required for the experiment are forint reagent, phenol, petroleum ether, ethyl acetate, sodium acetate, n-butanol, 95% ethanol, sodium carbonate, sodium nitrite, aluminum nitrate, sodium hydroxide and sulfuric acid purchased from Sinopharm Chemical Reagent Co., Ltd. Acetonitrile was purchased from DIKMA Technologies. Formic acid was purchased from TCI (Shanghai) Development Co., Ltd. 1,1-Diphenyl-2-trinitrophenylhydrazine (DPPH), 2,2-binitro-bis (3-ethyl-benzothiazole-6-sulfonic acid) diammonium salt (ABTS) and tert-butyl hydroperoxide (t-BHP) were purchased from Shanghai McLean Biochemical Technology Co., Ltd., China. High glucose DMEM medium was purchased from Gibco, USA. Ammonium format and ascorbic acid (vitamin C) obtained from Sigma-Aldrich. The 3-(4,5-dimethylthiazole-2)-2,5-diphenyltetrazole bromide (MTT) kit, 8-hydroxydeoxyguanosine (8-OHdG) kit and ROS kit were purchased from Shanghai Enzyme Link Biotechnology Co., Ltd., China. Interleukin-1β (IL-1β) and tumor necrosis factor-α (TNF-α) kits were purchased from Wuhan Boster Bioengineering Co., Ltd., China. All the agents were subjected to preliminary tests to confirm their suitability for this study before being used in the formal experiments.

Bi-distilled deionized water system (Laboratory Water Purification System, Shanghai Hitech Instrument Co., Ltd.), Electronic balances (Electronic balance PX224ENH, OHAUS Instruments GmbH), carbon dioxide incubator (HF90 carbon dioxide incubator, Shanghai Heal Force Bio-Meditech Holdings Co., Ltd.), inverted biological microscope (BDS500 Inverted Biological Microscope, Chongqing Optec Instrument Co., Ltd.), ELISA microplate analyzer (DNM-9602G microplate analyzer, Beijing Perlove New Technology Co., Ltd.), rotary evaporator (RE-2000A Rotary Evaporator, Shanghai Yarong Biochemical Instrument Factory), ultrasonic extractor (SB25-12DTD Ultrasonic Cleaner, Ningbo Xinzhi Biotechnology Co., Ltd.) and freeze dryer (SCIENTZ-10N Freeze Dryer, Ningbo Xinzhi Biotechnology Co., Ltd.) were used in this study.

### Experimental protocol

**Figure 2:**
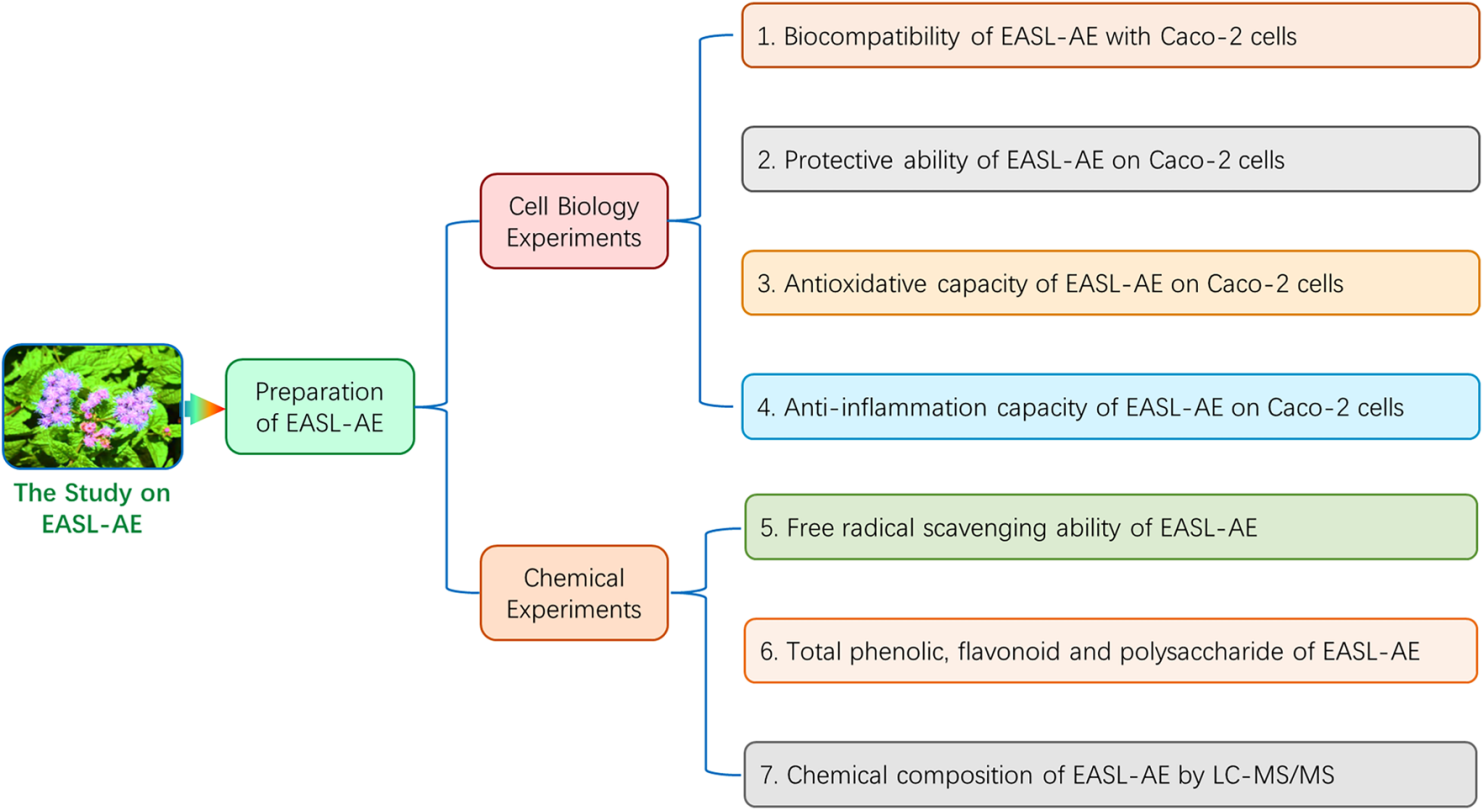
The experimental protocol of this study.

The experimental protocol of this study is shown in Fig. 2.

### Preparation of experimental samples

**Figure 3:**
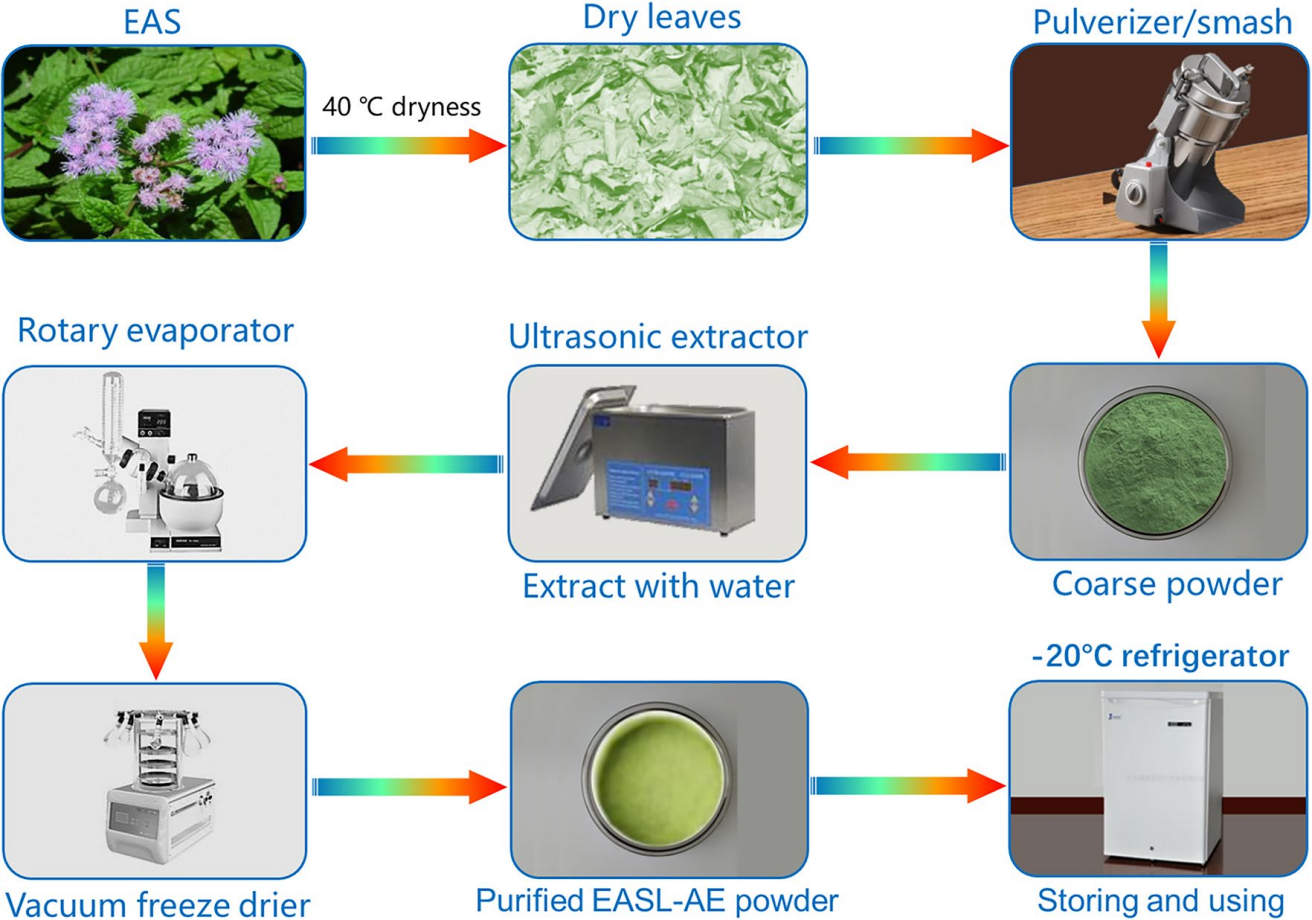
The extraction process of EASL-AE.

The EASL was collected after getting permission from departmental ethical committee following the protocol described by the Ernst 1995^29^. The leaves of EAS (EASL) were collected from Cangshan Erhai National Nature Reserve during the summer of 2022, and identified by Senior Botanical Taxonomist Li Ji-Hong from the Institute of Eastern-Himalaya Biodiversity Research, Dali University. After drying at 40 °C, the leaves were pulverized, and passed through a 100-mesh sieve. A 500 mL conical flask was used to weigh 50 g of EASL powder. Then, 500 mL of petroleum ether was added to achieve a material-liquid ratio of 1:10, and the mixture was subjected to ultrasonic sonication for 1 h at 20 °C, 60% power, and 40 KHz. Afterward, it was placed in a dark environment for 24 h. Filtration was then performed. The filtrate was concentrated using rotary evaporation and then lyophilized to obtain the petroleum ether extract of EASL. The filtered residue was dried at 40 °C and the same process was repeated using ethyl acetate, n-butanol, 95% ethanol and distilled water as extraction reagents to obtain the ethyl acetate extract, n-butanol extract, 95% ethanol extract and aqueous extract of EASL (EASL-AE), respectively. All extracts were stored at -20 °C (see Fig. 3).

### Cell line and its cultivation

Caco-2 cells (human colon adenocarcinoma cells) were obtained from the Kunming Typical Culture Cell Bank of the Chinese Academy of Sciences (KCB 200710YJ). Cell line serial numbers were checked for eligibility by ICLAC (The International Cell Line Authentication Committee) prior to formal experiments^30^. Inoculate cells into a 75 cm^2^ cell culture flask containing 15 mL of medium and place in a 37 ℃, 5% CO_2_, high humidity incubator. The medium was changed every 24 h. The medium consisted of 84% high glucose DMEM medium, 15% fetal calf serum, 1% penicillin (1000 U/mL) -1% streptomycin (1000 µg/mL). After the cells cover 80% to 90% of the bottom area of the bottle, they were sub-cultured^28^.

### Determination of biocompatibility of EASL-AE with Caco-2 cells

The biocompatibility of EASL-AE with Caco-2 cells was determined by 3-(3,5-dimethylthiazol-2-yl)-2,5-diphenyl-terazolium bromide (MTT) assay and lactate dehydrogenase (LDH) release. Caco-2 cells were inoculated into 96-well cell culture plates at 1 × 10^5^ cells per well, and after 2 days of culture, the cells were treated with different concentrations of EASL-AE (0.156, 0.31, 0.625, 1.25, 2.5, 5.0, 10.0 mg/mL) for 24 h. Cell viability (%) was determined using a commercial kit (Cat. No. 16H12B56, Shanghai Enzyme Link Biotechnology Co., Ltd., Shanghai, China), LDH was determined using an ELISA commercial kit (Catalog No. A020-2–2, Nanjing Jiancheng Bioengineering Institute Co., Ltd, Nanjing, China). All samples were prepared according to the MTT kit instructions. Absorbance was measured at 450 nm using a microplate reader^31^.

### Determination of protective ability of EASL-AE on Caco-2 cells

The biocompatibility test demonstrated the protective effect of EASL-AE on t-BHP-induced oxidative stress in Caco-2 cells. Furthermore, the results at 10 mg/mL were slightly higher than the control. Based on this, to save costs in subsequent experiments, the drug concentration was adjusted to 0.0, 1.0, 5.0, 10 mg/mL.

The concentration of Caco-2 cells was adjusted to 5 × 10^5^ cells/mL, seeded into 6-well plates with 2.0 mL per well, and cultured in a high humidity incubator at 37 °C and 5% CO_2_. The culture medium was changed every 12 h. After 2 days, discard the cell culture medium, add 1 mL of PBS into each well to wash the cells, and repeat 2 times. Then, Caco-2 cells were pre-protected with different concentrations (0.0, 1.0, 5.0, 10 mg/mL) of EASL-AE and 1 mg/mL of VC for 12 h, with 6 replicates in each group. Cells were then treated with 2 μmoL/mL t-BHP for 5 h. EASL-AE, t-BHP and VC were diluted in DMEM medium. Cell viability was measured by MTT kit (No. 16H12B56, Shanghai Enzyme Chain Biotechnology Co., Ltd, China), and all operations were performed according to the kit instructions. Absorbance was measured at 490 nm using an enzyme marker (DNM-9602G microplate analyzer, Beijing Perlove New Technology Co., Ltd.).

### Determination of antioxidative and anti-inflammation capacity of EASL-AE

ROS and 8-OHdG were used as indicators to evaluate the effect of EASL-AE on the ability of Caco-2 cells to resist oxidative stress/damage, while TNF-α and IL-1β were used as indicators to evaluate the effect of EASL-AE on the anti-inflammatory abilities of Caco-2 cells. The concentration of Caco-2 cells was adjusted to 5 × 10^5^ cells/mL, seeded into 6-well plates, 2.0 mL per well, and incubated in a high humidity incubator at 37 °C and 5% CO_2_. The culture medium was changed every 12 h. After 2 d, were discarded the cell culture medium, and add 1 mL of PBS was added into each well to wash the cells, repeating this process twice. Subsequently, the Caco-2 cells were pre-protected for 12 h with different concentrations (0.0, 1.0, 5.0, 10 mg/mL) of EASL-AE or 1 mg/mL of VC. Six replicates were performed for each group. Then, the cells were then treated with 2 μmoL/mL t-BHP for 5 h. EASL-AE, t-BHP and VC were diluted with DMEM medium. After 5 h, 200 μL of culture solution was aspirated from each well and transferred into a 1.5 mL centrifuge tube, sealed, and stored at -20 °C for the determination of ROS, 8-OHdG, NFκB, TNF-α and IL-1β^28^. Oxidative stress indicators, ROS, 8-OHdG and NFκB in Caco-2 cells were determined at 450 nm using ROS assay kit, 8-OHdG assay kit and NFκB assay kit (Catalog No. 202204 , Catalog No. 02/2022 and Catalog No. YJ345112, Enzyme-linked Biotechnology Co., Ltd., Shanghai, China). The oxidative inflammatory indicators, TNF-α and IL-1β, in Caco-2 cells were measured at 450 nm using TNF-α and IL-1β assay kits (Catalog No. 2391811208 and Catalog No. 1141816208, Boster Bioengineering Co., Ltd., Wuhan, China).

### Free radical scavenging ability of EASL-AE: DPPH Assay

The DPPH radical scavenger experiment was performed according to a previous report^32^. The DPPH were diluted with absolute ethanol to obtain different concentrations (1, 0.5, 0.25, 0.125, 0.0625, 0.03125, and 0.01562 mg/mL). The 50 mg/mL DPPH solution is absolute ethanol was mixed with different concentrations of EASL extracts at a ratio of 1:1 and left at 37 °C for 30 min in the dark. Absolute ethanol was used as the control. The absorbance of the reaction mixtures were recorded at 517 nm. Each sample had 5 replicate, 1 control and 1 blank control. In addition, the absorbance of DPPH at concentration of 5, 10, 20, 40, 60, 80 and 100 mg/L was measured at 517 nm, and a regression curve of its concentration versus absorbance was plotted as a standard curve to calculate the free radical scavenging rate of the samples. The formula for calculating the scavenging activity of DPPH free radicals by EASL extract was as follows:

$$\mathrm{DPPH\, radical\, scavenging\, activity}=(1-\frac{\mathrm{Abs\, of\, sample}-\mathrm{Abs\, of\, control}}{\mathrm{Abs\, of\, blank}})\times 100\mathrm{\%}$$

### Free radical scavenging ability of EASL-AE: ABTS assay

The ABTS radical scavenging test was performed according to a previous report^33^. The ABTS were diluted with absolute ethanol to obtain various concentrations (1, 0.5, 0.25, 0.125, 0.0625, 0.03125, and 0.01562 mg/mL). The ABTS solution in  absolute ethanol was mixed with different concentrations of EASL extracts at a 1:1 ratio and left at 37 °C for 30 min in the dark. Absolute ethanol was used as the control. The absorbance of the reaction mixtures was recorded at 734 nm. Each sample had 5 replicates, 1 control and 1 blank control. The formula for calculating the scavenging activity of ABTS free radicals by EASL extract was as follows:

$$\mathrm{ABTS\, radical\, scavenging\, activity}=(1-\frac{\mathrm{Abs\, of\, sample}-\mathrm{Abs\, of\, control}}{\mathrm{Abs\, of\, blank}})\times 100\mathrm{\%}$$

### Measurement of total phenolic, total flavonoid and polysaccharide content

The total phenolic content was quantified using Folin–Ciocalteu’s reagent with gallic acid as a standard^34^. Briefly, mix 1 mL of 1 mg/mL EASL-AE with 5 mL 10% Folin–Ciocalteu’s reagent in a test tube. After 3–8 min, 4 mL of 7.5% Na_2_CO_3_ was added. Mix well and allow to react for 1 h in the dark. The absorbance was measured at 765 nm. The total phenolic content is expressed in gallic acid equivalents (μg GAE/mg).

The total flavonoid content was quantified with rutin as the standard according to the reference method^35^. Briefly, 1 mL of 1 mg/mL EASL-AE was mixed with 600 µL of a 1:1 (v/v) mixture of 5% (w/v) NaNO_2_ and 10% Al (NO_3_)_3_., and the reaction proceeded as follows: After adding 1 mL of 1 mol/L NaOH and 3.4 mL of 30% ethanol mixed for 6 min, and allow the reaction to proceed in the dark for 15 min. Then measure the absorbance at 510 nm. Total flavonoid content is expressed in rutin equivalents (μg RE/mg).

Using rutin as a standard, and the total polysaccharide content was determined by the phenol–sulfuric acid method^36^. Briefly, 1 mL of 1 mg/mL EASL-AE was mixed with 1 mL of 5% phenol and 5 mL of 1.84 g/mL H_2_SO_4_. After reacting for 30 min in the dark, and the absorbance was measured at 490 nm. The total polysaccharide content is expressed in glucose equivalent (μg GE/mg).

### Chemical composition of EASL-AE by LC–MS/MS analysis

The freeze-dried EASL-AE material was sent to Suzhou Panomics Biomedical Technology Co. for LC–MS analysis. Please see Supp 1 for the detailed specific operational steps.

### Statistical analysis

GraphPad Prism 7.0 (San Diego, CA) was used to generate statistical graphs based on the experimental data. The values were presented as the mean ± SEM (standard error of the mean). To assess the significance of differences among groups, both analysis of variouce (ANOVA) and the least signification difference LSD tests were employed. A p-value of 0.05 was considered statistically significant.

## Results

### Biocompatibility of EASL-AE with Caco-2 cells

**Figure 4:**
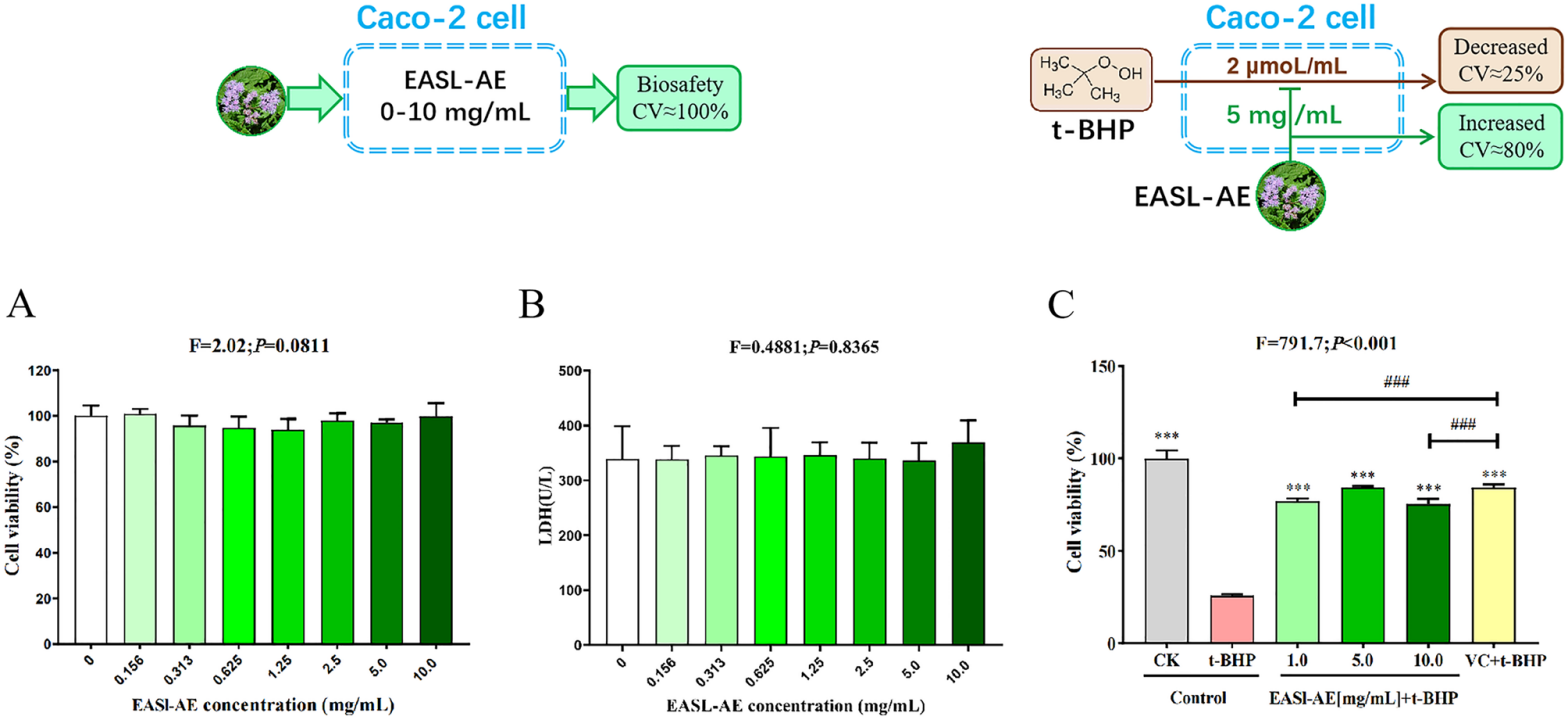
Cytocompatibility and Protective effect of EASL-AE. (**A**) MTT value (%) of different concentrations of EASL-AE; (**B**) LDH value (U/L) of different concentrations of EASL-AE; (**C**) Cytoprotective effect of EASL-AE on Caco-2 cells injured by t-BHP. (***: indicates *P* < 0.001, comparison with t-BHP group; ###: indicates *P* < 0.001, comparison with VC + t-BHP group).

To assess the biocompatibility, we treated Caco-2 cells with different concentrations of EASL-AE for 24 h and then evaluated the changes in cell viability and LDH release. The results showed that the cell viability under the effect of different concentrations of EASL-AE was changed slightly compared with that of the control group (0.00 mg/mL group), but there was no statistically significant difference (see Fig. 4A, F = 2.02, *P* = 0.0811), proving that EASL-AE had no influence on Caco-2 cells.

The results of LDH release detection (see Fig. 4B, F = 0.4881, *P* = 0.8365) showed that the LDH concentration under the effect of different concentrations of EASL-AE was slightly changed slightly compared with the control group (0.00 mg/mL group), but there was no statistically significant difference.

### Protective effect of EASL-AE on the Caco-2 cells with oxidative damage

After pre-protecting Caco-2 cells with different concentrations of EASL-AE (1.0, 5.0, 10.0 mg/mL) and 1 mg/mL VC for 12 h, the cells were subjected to oxidative stress by t-BHP at 2 μmol/mL. After 5 h, the cell viability of each group was measured using the MTT assay to assess the oxidative stress induced by t-BHP and the protective effect of EASL-AE against t-BHP-induced oxidative stress. The results (Fig. 4C, F = 791.7, *P* < 0.001) showed that after being treated with 2 μmoL/mL t-BHP, the viability of cells not pre-protected by VC or EASL-AE decreased to 25.65%, which was significantly lower than that of the control group (100%) (*P* < 0.001), indicating that t-BHP can cause severe cellular damage in Caco-2 cells; the cell viability in the VC + t-BHP group was 84.17%, which was significantly higher than that of the t-BHP group (*P* < 0.001), indicating that VC has a good protective effect on t-BHP-induced cell damage; When Caco-2 cells were pre-protected by different concentrations (1.0, 5.0, 10.0 mg/mL) of EASL-AE for 12 h before t-BHP oxidative stress treatment, and cell viability was 69.01%, 78.86% and 66.85% respectively, significantly higher than that of the t-BHP group (*P* < 0.001), indicating that EASL-AE treatment had a positive protective effect on cells.

### Anti-oxidative stress capacity of EASL-AE on Caco-2 cells

**Figure 5:**
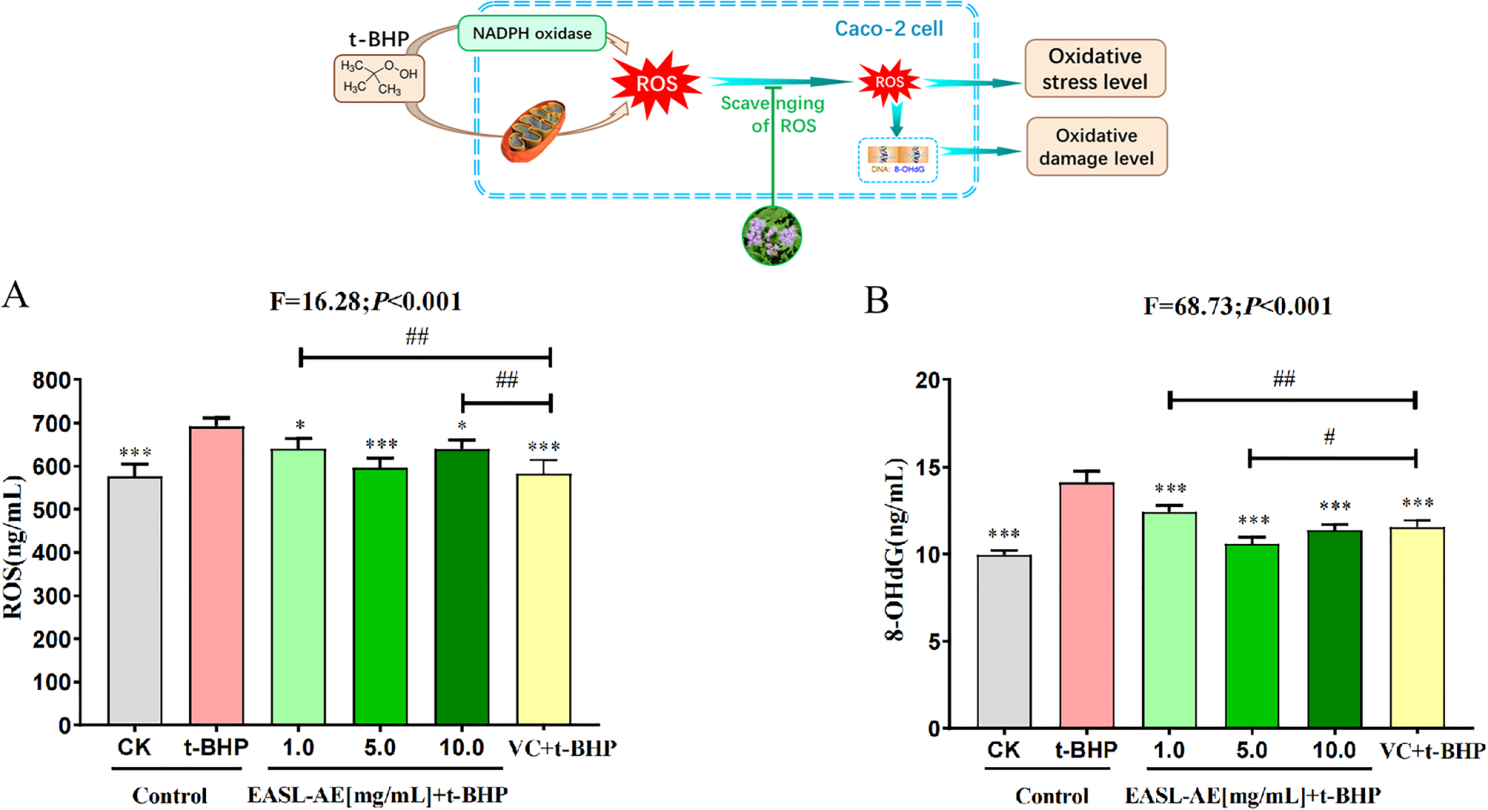
Antioxidative stress capacity of EASL-AE on Caco-2 cells. (**A**) Detection of cellular ROS level; (**B**) Detection of cellular 8-OHdG level; (*: *P* < 0.05, **: *P* < 0.01, ***: *P* < 0.001, comparison with t-BHP group; #: *P* < 0.05, ##: *P* < 0.01, comparison with VC + t-BHP group).

The results (Fig. 5A, F = 16.28, *P* < 0.001) showed that after 5 h of treatment with 2 μmol/mL t-BHP treatment for 5 h, the ROS content in Caco-2 cells increased significantly by 20.11% (*P* < 0.001) compared with the control group, indicating that t-BHP treatment induced the formation of oxidative stress in Caco-2 cells; After the cells were pre-protected by VC for 12 h and the treated with 2 μmol/mL t-BHP treatment, the ROS content was significantly reduced by 93.92% (*P* < 0.001) compared with that in the t-BHP group (*P* < 0.001), indicating that VC has good antioxidant activity; When the cells were pre-treated with different concentrations (1.0, 5.0, 10. 0 mg/mL) of EASL-AE for 12 h and then treated with 2 μmol/mL t-BHP, the ROS content of the cells in each group was significantly decreased by 43.92%, 82.75% and 43.92% , respectively, compared with that in the t-BHP group (*P* < 0.05, *P* < 0.001, *P* < 0.05), confirming that EASL-AE is an effective ROS scavenger. However, the ROS level in the 10 mg/mL EASL-AE-treated group was higher than that of the 5 mg/mL EASL-AE-treated group, suggesting that higher doses of EASL-AE may have some adverse effect on the cells, which must be account in the application.

The results (Fig. 5B, F = 68.73, *P* < 0.001) showed that after 2 μmoL/mL t-BHP treatment for 5 h, the level of 8-OHdG in Caco-2 cells increased significantly by 41.93% compared with the control group (*P* < 0.001), indicating that t-BHP treatment can induce oxidative stress in Caco-2 cells; Compared with the t-BHP group, the 8-OHdG content of the cells pre-protected with VC for 12 h and then t-BHP treatment was significantly decreased by 40.66% (*P* < 0.001), suggesting that VC has a good protective effect on t-BHP-induced DNA damage; Compared with the t-BHP group, the 8-OHdG content of the cells in the three groups pre-protected for 12 h with different concentrations (1.0, 5.0, and 10.0 mg/mL) of EASL-AE followed by t-BHP treatment was significantly lower than that of the t-BHP group, with decreases of 84.25%, 65.77%, and 61.66%, respectively (*P* < 0.001), and the performance the group pre-protected with 5.0 mg/mL EASL-AE was significantly better than that of the group pre-protected with VC in preventing DNA damage in Caco-2 cells caused by t-BHP (*P* < 0.05), This suggests that EASL-AE is a potent antioxidant compound against DNA damage. On the other hand, the 8-OHdG content of the 10 mg/mL EASL-AE pre-protected group was higher than that of the 5 mg/mL EASL-AE-treated group. It suggests that high doses of EASL-AE may have negative effects on cells, which should be carefully considered in its application.

## An-inflammatory capacity of EASL-AE on Caco-2 cells

**Figure 6:**
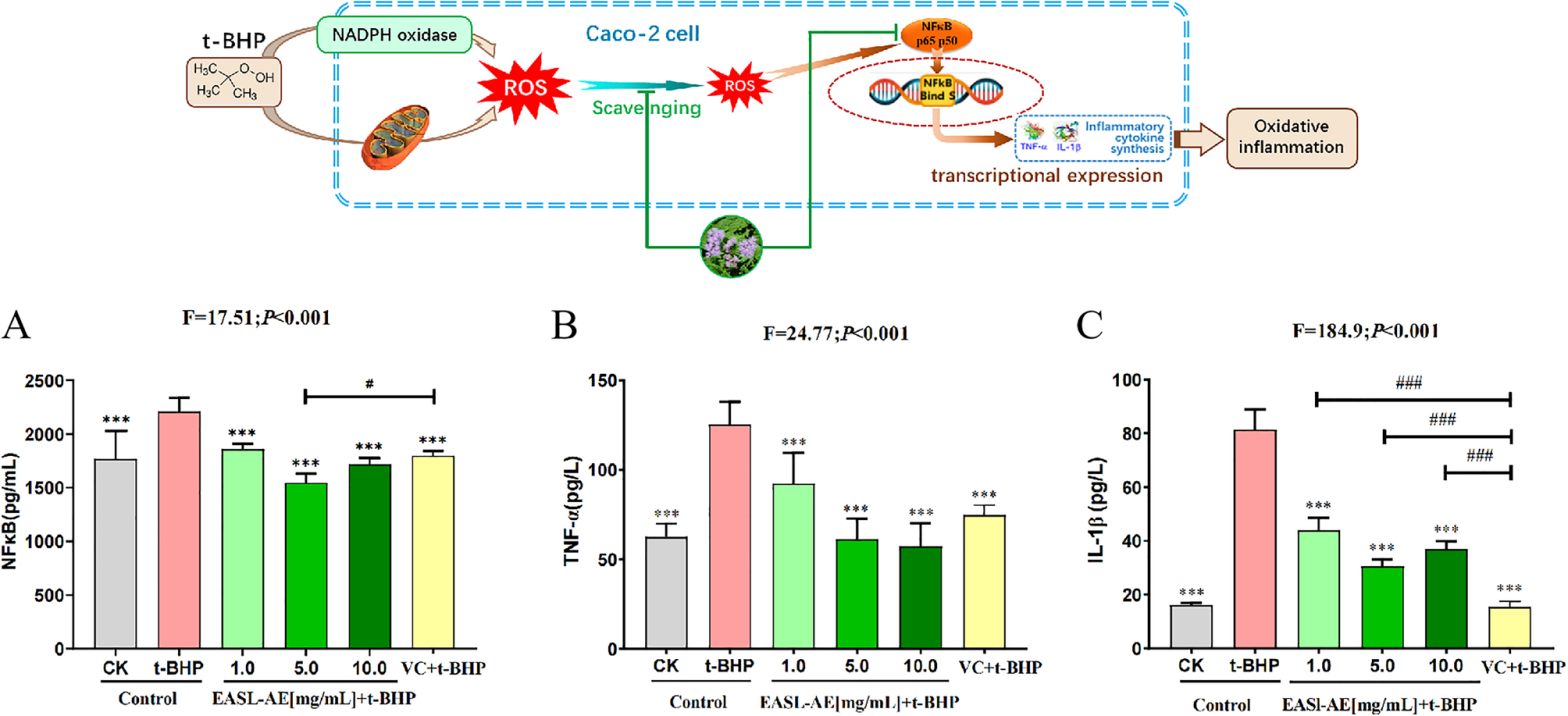
Anti-inflammatory capacity of EASL-AE on Caco-2 cells. (**A**) Detection of cellular NFκB level; (**B**) Detection of cellular TNF-α level; (**C**) Detection of cellular IL-1β level. (***: indicates *P* < 0.001, comparison with t-BHP group; #: indicates *P* < 0.05, ###: indicates *P* < 0.001, comparison with VC + t-BHP group).

The results (Fig. 6A, F = 17.51, *P* < 0.001) showed that after being treated with 2 μmoL/mL t-BHP for 5 h, the NFκB level in Caco-2 cells was significantly increased by 24.94% compared with that of the blank control group (*P* < 0.001), indicating that t-BHP treatment upregulates NFκB in Caco-2 cells, thereby triggering an inflammatory response; After the cells were pre-protected with VC for 12 h and then treated with 2 μmoL/mL t-BHP, their NFκB content was significantly reduced by 18.60% compared with that of the t-BHP group (*P* < 0.001), indicating that VC is a good anti-inflammatory agent; After the cells were pre-protected with different concentrations (1.0, 5.0 and 10.0 mg/mL) of EASL-AE for 12 h, and then treated with 2 μmoL/mL t-BHP, the level of NFκB in each group, was significantly decreased by 15.89%, 30.03% and 22.30% compared with the t-BHP group (*P* < 0.01, *P* < 0.001, *P* < 0.01), and that of the group pre-protected with 5.0 mg/mL EASL-AE was significantly better than that of the group pre-protected with VC in upregulated NFκB in Caco-2 cells caused by t-BHP (*P* < 0.05), indicating that EASL-AE achieves the effect of reducing the inflammatory response induced in cells by t-BHP by down-regulating the NFκB pathway. On the other hand, the NFκB content of the 10.0 mg/mL EASL-AE pre-protected group was higher than that of the 5.0 mg/mL pre-protected group. This suggests that high doses of EASL-AE may have negative effect on cells, but there was no statistically significant difference, and this should considered in the application.

The results (Fig. 6B, F = 24.77, *P* < 0.001) showed that after 2 μmol/mL t-BHP treatment for 5 h, the TNF-α content in Caco-2 cells significantly increased by 100.18% compared with the control group (*P* < 0.001), indicating that t-BHP treatment induced oxidative inflammatory responses in Caco-2 cells; After the cells were pre-protected by VC for 12 h followed by 2 μmol/mL t-BHP treatment, the TNF-α content was significantly reduced by 80.66% compared with the t-BHP group (*P* < 0.001), indicating that VC is a good anti-inflammatory agent; After the cells were pre-treated with different concentrations (1.0, 5.0 and 10.0 mg/mL) of EASL-AE for 12 h and then treated with 2 μmol/mL of t-BHP, the TNF-α content of the cells in each group was significantly decreased by 52.69%, 102.15% and 103.25% (*P* < 0.01, *P* < 0.01, *P* < 0.001), and there was no significant difference between the TNF-α content and that of the VC group, indicating that EASL-AE could effectively reduce the cellular inflammatory response induced by t-BHP, and that the 5.0 mg/mL and 10.0 mg/mL of EASL-AE inhibited t-BHP-induced oxidative inflammation in Caco-2 cells better than VC, suggesting that EASL-AE is a very promising anti-inflammatory agent.

The results (Fig. 6C, F = 184.9, *P* < 0.001) showed that after being treated with 2 μmoL/mL t-BHP for 5 h, the IL-1β level in Caco-2 cells was significantly increased by 502.27% compared with that of the blank control group (*P* < 0.001), indicating that t-BHP treatment caused the inflammatory response in Caco-2 cells; After the cells were pre-protected with VC for 12 h and then treated with 2 μmoL/mL t-BHP, their IL-1β content was significantly reduced by 101.05% compared with that of the t-BHP group (*P* < 0.001), indicating that VC is a good anti-inflammatory agent; After the cells were pre-protected with different concentrations (1.0, 5.0 and 10.0 mg/mL) of EASL-AE for 12 h, and then treated with 2 μmoL/mL t-BHP, the level of IL-1βin each group, was significantly decreased by 57.54%, 77.88% and 68.20% compared with the t-BHP group (*P* < 0.001, *P* < 0.001, *P* < 0.001), (although significantly higher than the VC + t-BHP group (*P* < 0.001)), indicating that EASL-AE could effectively reduce the inflammatory response in cells induced by t-BHP. The 10 mg/mL EASL-AE-treated group had higher IL-1β levels than the 5 mg/mL EASL-AE-treated group, indicating high doses of EASL-AE may have certain effects on cells, which should be considered when applying.

### Free radical scavenging activity of EASL-AE

**Figure 7:**
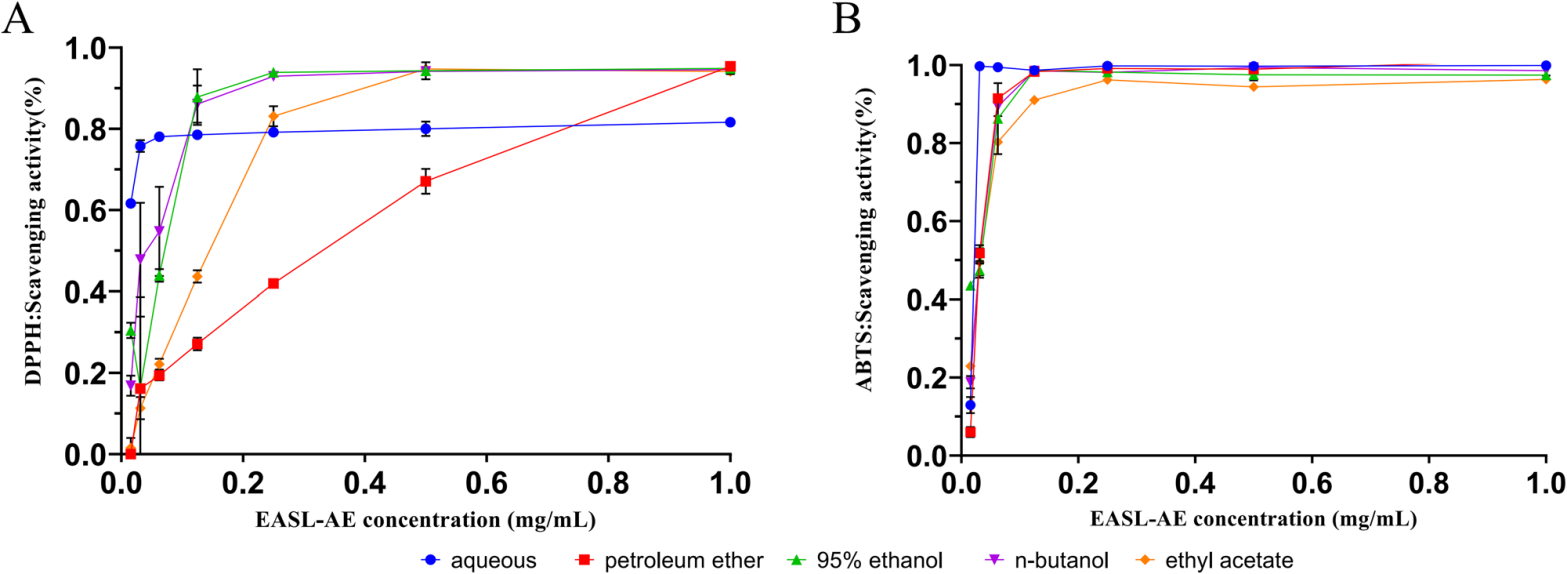
Antioxidant activity of EASL extracts. (**A**) Scavenging activity of different solvent extracts of EASL against DPPH; (**B**) Scavenging activity of different solvent extracts of EASL against ABTS.

The antioxidant activity of various solvent extracts was evaluated by DPPH and ABTS radical scavenging assays. The results showed that the scavenging ability of EASL extracts prepared with five solvents (petroleum ether, ethyl acetate, n-butanol, 70% ethanol and distilled water) on DPPH and ABTS free radicals exhibited a concentration dependent mode (Fig. 7). According to the IC50 values, the order of DPPH radical scavenging activity was n-butanol extract > aqueous extract > 95% ethanol extract > ethyl acetate extract > petroleum ether extract, while ABTS radical scavenging activity the order was aqueous extract > 95% ethanol extract > n-butanol extract > ethyl acetate extract > petroleum ether extract (Table 1). Taken together, the aqueous extract of EASL (EASL-AE) had the highest antioxidant activity.

**Table 1:** IC50 of different solvent extracts of EASL against DPPH and ABTS radicals.

Sample	IC50 (μg/mL)	
	DPPH assay	ABTS assay
EASL extract by aqueous	55.44	13.20
EASL extract by 95% ethanol	64.57	22.85
EASL extract by ethyl acetate	126.40	29.71
EASL extract by n-butanol	38.53	29.58
EASL extract by petroleum ether	257.20	30.92

Based on the following considerations, in the subsequent experiment, we will only test the antioxidant activity of EASL-AE: (1) among the EASL extracts obtained from different solvents, the aqueous extract of EASL (EASL-AE) had the highest free radical scavenging activity taken together; (2) in order to obtain the most accurate results for evaluating the antioxidant and anti-inflammatory activities of the extracts, it is desirable for the samples not to have been contaminated by the solvents; and in view of the practical applications in the later stage, it is desirable for the extracting reagents used to be commonly available and non-toxic; and (3) for the medicinal use of EASL, the main focus is on the use of its aqueous extract.

### Total phenolic, flavonoid and polysaccharide content of EASL-AE

In this study, the contents of polyphenols, flavonoids and polysaccharides of EASL-AE were determined, and the results showed that the contents of flavonoids were highest, followed by polysaccharides and polyphenols in EASL-AE (Table 2).

**Table 2:** Total phenolic, flavonoid and polysaccharide content of EASL-AE.

Chemical Com Position	Content (mean ± SD)
Total phenolic content (TPC) (μg GAE/mg)	112.68 ± 0.11
Total flavonoid content (TFC) (μg RE/mg)	481.21 ± 0.43
Total polysaccharide content (μg GE/mg)	160.41 ± 0.40

### Chemical composition of EASL-AE by LC–MS/MS analysis

**Figure 8:**
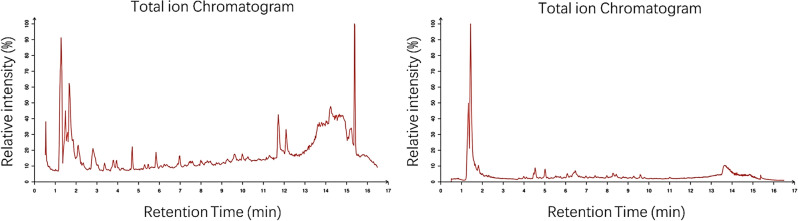
LC–MS analysis of EASL-AE. (Left): Positive ion flow diagram; (Right): Negative ion flow diagram.

The chemical composition of EASL-AE was analyzed by untargeted UPLC-MS/MS. A total of 222 compounds were identified, including 12 polyphenols, 11 amino acids, 4 flavonoids and 8 terpenoids, of which 159 were detected in the positive ion mode and 63 in the negative ion mode. It can be seen that EASL-AE has the highest content of flavonoids but the lowest number of compounds, while polyphenols have a lower content but the highest number of compounds. This suggests that the antioxidant activity of EASL-AE is attributable to multiple substances, including various flavonoids and polyphenols^37^. In addition, a variety of amino acids and terpenoids are also existed in EASL-AE, suggesting that EASL-AE may have a variety of unidentified biologically active functions (Fig. 8).

## Discussion

### Biosafety of EASL-AE and its protection on the Caco-2 cells with oxidative damage

Biosafety and efficacy are the basic requirements for biomaterials intended for medical and healthcare use^38,39^. Biosafety, also known as 'biocompatibility' or 'cytotoxicity', refers to the assessment of whether a drug or biomaterial will cause cell damage or impair cell function. The MTT assay is a commonly used method to test biocompatibility^40–42^. Previous reports have shown that EAS extracts were cytotoxic, such as the EASL ethanol extract to Hale cells^12^, the EASL methanol extracts to lung cancer cells (A549)^17^, and the EASL essential oil to HePG2, Hep3B, SMMC-7721, and LO2 cells^11^. However, there have been no studies on the cytotoxicity of EASL-AE on Caco-2 cells. Our study found that EASL-AE was non toxic to Caco-2 cells at experimental concentrations ranging from 0.0 to 10.0 mg/mL (see Fig. 4A). Additionally, there was no increase in LDH release from Caco-2 cells, indicating that EASL-AE of 0.0 to 10.0 mg/mL did not impose an additional burden on Caco-2 cells (see Fig. 4B). This suggests that the aqueous extract (AE) may be safer than other solvent extracts, and controlling the concentration of use may also be an important measure in ensuring biosafety. Furthermore, our study also found that EASL-AE treatment has a positive protective effect on Caco-2 cells subjected to oxidative damage (Fig. 4C), which is similar to the findings of other literature^43,44^.

Furthermore, our study indicated that the effectiveness of EASL-AE was not dose-dependent, hinting at a potential non-linear dose–response relationship. This may be attributed to the cytotoxic effects of EASL-AE itself, as cell toxicity of EAS extract has been documented in prior research^45,46^. This toxicity is largely due to compounds such as sesquiterpenes, including 9-oxo-10,11-dehydroagerophorone (euptox A), 2-deoxo-2-(acetyloxy)-9-oxo-ageraphorone (DAOA), and 9-oxoagerophorone (OA), identified as the primary toxic components^47^. Thus, we advise careful dosage control when utilizing EAS for its antioxidative properties, to mitigate potential risks.

### The mechanisms of t-BHP induced oxidative stress in Caco-2 cells

Oxidative stress is primarily caused by the excessive accumulation of intracellular reactive oxygen species (ROS). Any endogenous or exogenous factor that leads to the excessive accumulation of reactive oxygen species in the cell can result in oxidative stress^48,49^. T-BHP, a frequently utilized peroxide, has the ability to stimulate the generation of reactive oxygen species (ROS) within cells. The induction of ROS production by t-BHP primarily takes place through two main pathways: (1) Upon entering the cell, NADPH oxidase is activated, leading to ROS production^50^. (2) t-BHP interferes with the mitochondrial respiratory chain, resulting in ROS production. This is consistent with the situation described in our scientific hypothesis (see Fig. 1).

### The antioxidant stress and antioxidant damage effect of EASL-AE

In this study, we observed that EASL-AE effectively scavenged intracellular reactive oxygen species (ROS) (see Fig. 5A). Additionally, we found that extracellularly, EASL was able to scavenge DPPH and ABTS free radicals (see Fig. 7A,B). Among the different extracts, the aqueous extract demonstrated the strongest ability to scavenge ABTS, with an IC50 value of 13.20 μg/mL (see Table 1). The great potential application of the aqueous extracts was also demonstrated. Chemical analysis of EASL-AE revealed a high content of phenolic, ketone, and polysaccharide compounds (see Table 2). Specifically, EASL-AE exhibited a higher total flavonoid content of 481.21 ± 0.43 μgRE/mg compared to the total polysaccharide content of 160.41 ± 0.40 μgGE/mg, and the total phenolic content of 112.68 ± 0.11 μgGAE/mg. It is well-established that phenolics, flavonoids and polysaccharides present in plants have strong scavenging effects on free radicals and ROS^51–53^, thereby demonstrating excellent anti-oxidative stress properties.

The concepts of oxidative stress and oxidative damage are distinct. Oxidative stress is a state in which large amounts of ROS are produced in the cell when the environment inside and outside the cell changes, leading to a dysregulation of intracellular redox balance. Oxidative stress is a broader concept that primarily refers to the excessive buildup of intracellular reactive oxygen species (ROS) and the subsequent cascade of biochemical and pathological events^54,55^, including oxidative damage and oxidative inflammation. However, the fundamental molecular event in this process is the excessive accumulation of ROS^48,56^. On the other hand, oxidative damage specifically pertains to the harm inflicted on biomolecules by ROS^57,58^. This includes DNA damage resulting in the formation of 8-OHdG^59^, damage to membrane lipid molecules leading to the formation of MDA^60^, and harm to protein molecules resulting in protein carbonylation^61^. Our scientific hypothesis diagram (Fig. 1) demonstrates that exposure to t-BHP causes an excessive accumulation of ROS, which in turn leads to DNA damage and the production of 8-OHdG. Conversely, the use of EASL-AE clears ROS, thereby providing protection against DNA damage (8-OHdG) by reducing the amount of ROS generated.

### The anti-inflammatory effect of EASL-AE and its molecular mechanism

Inflammation caused by oxidative stress is commonly referred to as 'oxidative inflammation'^62,63^. Two key indicators of the severity of oxidative inflammation are TNF-α and IL-1β^64,65^. Under conditions of oxidative stress, the expression of these inflammatory factors is upregulated. They play crucial roles in the inflammatory response, immune response, and disease development. The intensity of oxidative inflammation is regulated by three mechanisms (see Fig. 1): (1) the level of intracellular ROS concentration. Higher concentrations of intracellular ROS lead to increased synthesis of oxidative inflammation end-products TNF-α and IL-1β, which is a ROS-dependent effect^66^. (2) the level of activation of the Nrf2 signaling pathway. The Nrf2 signaling pathway is an important intracellular pathway that plays a key protective role in regulating intracellular oxidative stress and antioxidant defense^67^. Nrf2, a transcription factor, regulates the expression of various antioxidant and detoxification enzymes, including GSH, SOD, and HO-1, to counteract oxidative stress and maintain intracellular oxidative homeostasis^68^. Therefore, higher activation of the Nrf2 signaling pathway results in lower production of inflammatory factors; (3) Activation level of the NFκB signaling pathway: NFκB, a transcription factor, plays a crucial role in cellular signaling and is closely associated with the regulation of inflammatory factors^69^. Upon stimulation by ROS, NFκB gets activated and translocated to the nucleus, leading to gene transcription and expression of inflammatory factors^70^. Inflammatory factors like TNF-α and IL-1β further enhance the activation of NFκB, establishing a positive feedback loop and intensifying the inflammatory response^71^. Thus, there exists an intricate interplay between the NFκB signaling pathway and inflammatory factors, which significantly contributes to the onset and progression of inflammatory diseases. In summary, higher activation of the NFκB signaling pathway correlates with increased risk of oxidative inflammation^72^.

This shows that the anti-inflammatory effect of EASL-AE is achieved through the three mechanisms mentioned above. EASL-AE scavenges intracellular ROS and reduces TNF-α and IL-1β concentration levels. Furthermore, our results (Fig. 6) showed that the use of 1.0–10.0 mg/mL EASL-AE significantly reduced NFκB signaling proteins, down-regulated the activation of the NFκB signaling pathway and consequently reduced the levels of t-BHP-induced inflammatory factors TNF-α and IL-1β. However, it is important to note that in this experiment, we did not measure the Nrf2 signaling-pathway related indexes to qualitatively or quantitatively assess the effect of EASL-AE on this pathway.

### EASL-AE with great development prospects for medicine and healthcare

In the present study, we made an exciting discovery. Through LC–MS/MS analysis, we identified 222 compound molecules in EASL-AE. Among the 10 most abundant organic molecules found in EASL-AE (Table 3), some exhibit antioxidant activity, such as gentianic acid, procaine, and L-tyrosine^73,74^. Additionally, certain molecules in EASL-AE demonstrate anti-inflammatory properties, including gentianic acid, loratadine, and 4-hydroxycoumarin^75–77^. Furthermore, some compounds in EASL-AE exhibit antimicrobial activity, such as gentianic acid, hydroquinone, and 4-hydroxycurcumin^78,79^. These findings suggest that EASL-AE possesses multiple biological activities and holds promise for further development.

**Table 3:** LC–MS analysis of EASL-AE*.

Name	Formula	m/z	RT (s)	ppm	pos/neg	X (Mean ± SD)
Gentisic acid	C_7_H_6_O_4_	154.99	957.2	2.32	Pos	7,689,443,175 ± 397,728,480
Procaine	C_13_H_20_N_2_O_2_	236.15	110	16.52	Pos	3,257,081,492 ± 155,599,608
L-Phenylalanine	C_9_H_11_NO_2_	166.08	282	7.95	Pos	2,350,453,276 ± 120,909,706
Acetyl Phos Phate	C_2_H_5_O_5_P	139.99	923.3	0.71	Pos	2,446,493,070 ± 161,144,413
Nore Pine Phrine	C_8_H_11_NO_3_	169.78	610.1	0	Pos	1,181,692,038 ± 816,223,065
Loratadine	C_10_H_17_N	152.14	884.2	0	Pos	1,707,144,967 ± 430,737,792
4,5-Dihydroorotic acid	C_5_H_6_N_2_O_4_	158.96	948.5	1.81	Pos	1,006,088,118 ± 330,926,839
Hydroquinone	C_6_H_6_O_2_	111.02	966.6	4.93	Pos	594,640,105 ± 131,741,843
L-Tyrosine	C_9_H_11_NO_3_	182.02	228	2.07	Pos	419,005,138 ± 23,597,121
4-Hydroxycoumarin	C_9_H_6_O_3_	163.04	539.2	0	Pos	186,723,803 ± 187,303,569

*: m/z: mass-to-charge ratio; RT: retention time; ppm: error between the detected molecular weight and the theoretical molecular weight in ppm; pos/neg: positive/negative; X (mean ± SD): mean ± standard deviation of the distribution of signals of the metabolite in a given type.

In China, EAS is considered a highly detrimental invasive alien species. Due to its vigorous growth and resilience, it can easily establish itself as an invasive species in new environments, leading to significant harm to local ecosystems. This harm includes exerting competitive pressure on soil, water resources, and native plants. However, if our scientific research can find ways to mitigate the negative impacts of this species, it holds the potential to create a mutually beneficial situation for both healthcare and ecological conservation, ultimately benefiting mankind.

### Limitations of this study

Although the results of this study adequately demonstrated the antioxidant and anti-inflammatory effects of EASL-AE and achieved our expected research objectives. However, there is still a shortcoming in this study that the corresponding indicators of the Nrf2 signaling pathway, such as Nrf2, HO-1, GSH and SOD, were not measured. Therefore, this study could not answer the question as to whether there is a meaningful synergistic effect of the Nrf2 signaling pathway in the antioxidant and anti-inflammatory processes of EASL-AE. In future similar studies, we will endeavor to avoid this shortcoming.

### Supplementary Information

Supplementary Information.

## Data Availability

Data is provided within the supplementary information files (As Supplementary files).

## Abbreviations

EAS: *Eupatorium adenophorum* Spreng
EASL: The leaves of *Eupatorium adenophorum* Spreng
EASL-AE: The aqueous extract of the leaves of *Eupatorium adenophorum* Spreng
NCDs: Chronic non-communicable diseases
DPPH: 1,1-Diphenyl-2-picrylhydrazyl radical 2,2-Diphenyl-1-(2,4,6-trinitrophenyl) hydrazyl
ABTS: 2,2'-Azino-bis(3-ethylbenzothiazoline-6-sulfonic acid)
LC–MS: Liquid Chromatograph Mass Spectrometer
Caco-2: Colon adenocarcinoma cells
VC: Vitamin C
t-BHP: Tert-butyl hydroperoxide
MTT: 3-(3,5-Dimethylthiazol-2-yl)-2,5-diphenyl-terazolium bromide assay
LDH: Lactate dehydrogenase
8-OHdG: 8-Hydroxydeoxyguanosine
ROS: Reactive oxygen species
TNF-α: Tumor necrosis factor-α
IL-1β: Interleukin-1β
NFκB: Nuclear factor κ-B
