# Supplementary material for: Antioxidant and anti-inflammatory function of Eupatorium adenophora Spreng leaves (EASL) on human intestinal Caco-2 cells treated with tert-butyl hydroperoxide
Source: Sci Rep. 2024 May 7;14:10509. doi: 10.1038/s41598-024-61012-7 (PMC11076498; doi:10.1038/s41598-024-61012-7)
Supplement: Supplementary file 1 — Supplementary Information. [file 41598_2024_61012_MOESM1_ESM.pdf]

LC-MS analysis

The freeze-dried *EASl-AE* material was sent to Suzhou Panomics Biomedical Technology Co. for LC-MS analysis. Dissolve the samples in water, adjust the concentration to 1 g/mL, centrifuge at 12,000 rpm at 4 °C for 10 min, and filter the supernatant with a 0.22 μm filter membrane to obtain the solution to be measured. For chromatographic detection of *EASl-AE,* an Ultra-High Performance Liquid Chromatography (UPLC) (ACQUITY, Waters, Milford, MA, USA) and Thermo Q Exactive (Thermo Fisher Scientific, USA) were used for mass spectrometry. The ion source was electrospray ionization (ESI). Chromatographic and mass spectrometry were performed in positive and negative ion modes, respectively.

Chromatographic conditions: ACQUITY UPLC® HSS T3 column (2.1×150 mm, 1.8 μm) (Waters, Milford, MA, USA), flow rate of 0.25 mL/min, column temperature of 40 ℃, injection volume of 2 μL. In positive ionization mode, the mobile phases consists of 0.1% formic acid-acetonitrile (C) and 0.1% formic acid-water (D), and the elution gradient is as follows: 0~1 min, 2% C; 1~9 min, 2%~50% C; 9~12 min, 50%~98% C; 12~13.5 min, at 98% C; 13.5~14 min, 98%~2% C; 14~20 min, 2% C. In the negative ionization mode, the mobile phases were acetonitrile (A) and 5 mmol/L ammonium formate aqueous solution (B) gradient elution, the elution program was: 0-1 min, 2% A; 1-9 min, 2%-50% A; 9-12 min, 50%-98% A; 12-13.5 min, 98% A; 13.5-14 min, 98%-2% A; and 14-17 min, 2% A[S1].

The operating parameters of the mass spectrometry positive ion spray voltage of 3.50 kV, negative ion spray voltage of -2.50 kV, sheath gas of 30 Arb, auxiliary gas of 10 Arb, capillary temperature of 325 ℃, the primary full scan resolution of 70,000, and ion scan range of m/z 81~1000, and the HCD was used to perform the secondary fission, the collision voltage is 30% and the secondary resolution was 17500, The first 10 ions of the acquired signal were fragmented and unnecessary MS/MS information was removed by dynamic exclusion[S2].

Supplementary table 1: LC-MS analysis of EASL-AE

Name	Formula	m/z	RT (s)	ppm	pos/neg	**X-1**	**X-2**	**X-3**
Gentisic acid	C7H6O4	154.9896	957.2	2.320909255	pos	7464888842	8148663359	7454777324
Procaine	C13H20N2O2	236.1486	110	16.51	pos	3407	32668	30968

				502486		579814	26031	38630
Acetylphosphate	C2H5O5P	139.9876	923.3	0.714348985	pos	2263947991	2568998482	2506532737
L-Phenylalanine	C9H11NO2	166.0847	282	7.954577827	pos	2480592328	2329167621	2241599878
Loratadine	C10H17N	152.1434	884.2	0	pos	1221361077	1857594743	2042479081
Norepinephrine	C8H11NO3	169.9776	610.1	0.000574523	pos	1346276577	1903081322	295718216.4
4,5-Dihydroorotic acid	C5H6N2O4	158.9615	948.5	1.810764223	pos	625494187.5	1225944648	1166825518
Hydroquinone	C6H6O2	111.0212	966.6	4.929049623	pos	514395616.6	746684329.3	522840367.9
Guanidoacetic acid	C3H7N3O2	116.9257	890.5	13.31306492	neg	389703969.9	441479578.4	426375051
L-Tyrosine	C9H11NO3	182.0808	228	2.065017289	pos	446222747.7	406503309.4	404289357
L-Tryptophan	C11H12N2O2	205.0977	328.6	0.00030309	pos	334985100.1	300187423.1	300281763.6
2-Ketobutyric acid	C4H6O3	102.0345	437.6	2.595253586	pos	84340281.7	405207039.7	358185028.7
Dodecanoic acid	C12H24O2	199.9762	960.6	5.359768672	pos	301595473.5	309210284.5	231678475.1
Methyloxaloacetate	C5H6O5	147.0289	171.2	0.004930861	pos	250846197	293216505.7	287710854.3
2'-Deoxyguanosine	C10H13N5O4	266.9374	925.7	1.060568766	pos	229147423.7	254169740.9	256654596.9
4-Oxoproline	C5H7NO3	130.0496	927.2	2.122267197	pos	290951930.6	219513870.2	208050056.2

Atractylenolide II	C15H20O2	233.1535	559.2	0.454636109	pos	198299368.1	198836087.5	219546498.5
Kynurenic acid	C10H7NO3	190.0497	345.4	0.926073548	pos	208380903	192784620	203408848.1
Ciliatine	C2H8NO3P	125.9866	371.5	0.280694693	pos	117557571.6	233735273.8	236659240.2
4-Hydroxycoumarin	C9H6O3	163.0392	539.2	0.007949714	pos	403003043.5	78891654.35	78276709.98
2,3-Butanediol	C4H10O2S2	154.9895	660.7	4.3777677	pos	74789270.69	210356665	255989767.7
Epsilon-caprolactam	C6H11NO	114.0915	898	0.006412371	pos	27266029.34	26260716.61	484703560.7
Uracil	C4H4N2O2	113.0348	141	0.009415304	pos	182667340	176805353.2	177174593.8
L-Threonine	C4H9NO3	120.0655	90.5	0.199890893	pos	167103071	186718705.8	171010266.1
Phthalic acid	C8H6O4	149.0219	640.5	18.34629675	pos	46783861.03	248263639.7	209554999.1
Thymine	C5H6N2O2	127.0505	275.8	1.294763405	pos	162989559.1	158015336.8	149248660.8
Fructose 1,6-bisphosphate	C6H14O12P2	339.1992	885.2	1.585549833	neg	141240512.9	159297679.3	135565342.1
Phenyllactate	C9H10O3	165.0534	301.6	14.08029159	neg	212662536.7	82196565.76	86748765.54
Quinolinic acid	C7H5NO4	167.0134	247.9	1.871554049	pos	171365110	97627968	105794104.4
Anabasine	C10H14N2	163.0392	730.2	0.001048202	pos	100995445.6	42694683.73	229334336.4
cis-Aconitic acid	C6H6O6	157.0134	171.2	28.239627	pos	10179795	128021494.	121030071.

				96		6.6	3	3
Pyroglutamic acid	C5H7NO3	130.0509	637.7	4.516561024	pos	109075220	136369937.1	104515792.5
O-Acetylserine	C5H9NO4	148.0613	123.7	5.565262496	pos	116642292.6	111699820	112870352.7
2-Keto-6-acetamidocaproate	C8H13NO4	188.0913	179.6	2.530685896	pos	105596134.4	121704351.8	101920218.5
L-Asparagine	C4H8N2O3	132.103	726.4	7.878484849	pos	84495789.42	183394943.3	56023499.49
Nicotinic acid	C6H5NO2	122.5475	584.8	2.003377711	pos	144942268.5	32033186.04	135490703.9
Capsidiol	C15H24O2	219.1749	544.7	0	pos	63967993.35	59102626.64	187479309.1
Geranyl diphosphate	C10H20O7P2	314.088	234	3.89699702	pos	105838071.5	96483323.37	101257865.7
4-Hydroxycinnamic acid	C9H8O3	165.0563	228	6.182529749	pos	105791272.2	95886419.81	94995070.11
L-Proline	C5H9NO2	116.0712	690.6	0.391623366	pos	114745158.6	148348168.7	32240526.89
Estragole	C10H12O	149.096	533.3	0.046667972	pos	91629557.59	83574584.65	94837827.15
N-Carbamoylputrescine	C5H13N3O	132.103	681.5	5.481462298	pos	63681095.47	126656722.3	76414386.11
Pterin	C6H5N5O	163.0507	932.8	7.972980184	pos	82960776.04	106875941.4	73705767.93
(2E)-Decenoyl-ACP	C6H11NO2	130.0861	790.2	1.188382156	pos	63338351.09	79328619.32	106317037.6
Isoalantolactone	C15H20O2	233.1541	524.4	2.186607939	pos	80031155.72	75984381.14	83389448.67
Guanidinosu	C5H9N3O4	174.9531	446.7	9.649	ne	6715	12727	35004

ccinic acid				488966	g	3129.57	6901.6	096.29
Catechol	C6H6O2	111.0207	565.2	0.949002186	pos	45989574.28	69333042.9	113179249.7
Hypoxanthine	C5H4N4O	137.0466	38	1.55626887	pos	38797128.14	126452247	53463362.45
Salicylic acid	C7H6O3	137.0226	412.1	2.695499983	neg	17498012.78	39124672.51	159438462.3
Pimelic acid	C7H12O4	159.9696	148.3	2.928870692	pos	78674814.52	109399704	23453363.03
Pipecolic acid	C6H11NO2	130.086	184.6	2.121673354	pos	10571741.04	116157698.3	84588898.7
L-Isoleucine	C6H13NO2	132.103	296.3	5.208741066	pos	71113827.13	64691060.66	69500120.37
Caryophyllene epoxide	C15H24O	203.179	407.9	18.37788354	pos	65605013.02	62222565.98	69337832.91
Cyclohexylamine	C6H13N	100.1132	252.4	11.22729071	pos	108264191.4	25893219.47	61522072.06
Xanthine	C5H4N4O2	153.041	205.1	0.333061212	pos	62942315.28	62479430.56	63980984.85
3-Indoleacetonitrile	C10H8N2	156.1207	711.1	0.727211087	pos	35130447.6	106054829.1	43973646.35
Costunolide	C15H20O2	233.1537	599.4	0.403682206	pos	49653657.83	43103746.45	88500124.39
Dihydrouracil	C4H6N2O2	112.9846	875.7	1.248636186	neg	35081285.84	65777117.41	71125288.88
2'-O-Methyladenosine	C11H16N5O7P(C5H8O6PR)n(C5H8O6PR)n	282.1202	260.1	1.772294221	pos	57505999.51	52056949.53	54306715.55
4-Hydroxybenzoic acid	C7H6O3	137.024	558.1	2.919196637	neg	47780187.11	60700046.19	54267728.69

1,2-Epoxy-p-menth-8-ene	C10H16O	153.1283	462.6	5.877424356	pos	56298727.98	53942639.62	50325705.25
Mannitol	C6H14O6	182.0824	184.1	6.357594661	pos	55754989.92	52692202.44	50695026.66
Dibutyl phthalate	C16H22O4	279.1547	594.2	15.67589584	pos	27453388.03	55599033.37	72319644.05
Cytosine	C4H5N3O	112.0509	94.3	1.359789453	pos	54909247.99	49695161.37	50029361.43
Heptanoic acid	C7H14O2	130.1594	602.4	0.051394314	pos	53793676.33	43033216.31	55967297.38
Toluate	C8H8O2	135.0431	497	14.9878076	neg	43673875.53	26846700.87	78675500.38
Sebacic acid	C10H18O4	185.114	619.3	1.557581155	pos	46469111.05	65608955.79	33020567.84
2,6-Diethylaniline	C10H15N	149.1167	806.7	24.81278086	pos	12778521.62	49747537.19	78785763.75
L-Histidine	C6H9N3O2	155.0827	256.9	21.43230223	pos	47347999.85	47520563.81	44708933.85
Cycloheximide	C15H23NO4	282.1707	345.4	2.565822745	pos	50859784.43	45466173.6	42369273.66
Nootkatone	C15H22O	201.163	555.6	16.57362437	pos	65910812.06	31599111.83	37607006.16
2-Keto-glutaramic acid	C5H7NO4	145.0499	98.4	0.951955375	pos	32044934.25	64766803.11	33554804.04
4-Hydroxyphenylacetaldehyde	C8H8O2	136.0616	308.3	0.075369325	pos	41937335.65	43251277.24	42022325.54
Curcumenol	C15H22O2	217.1597	473.5	23.64158727	pos	33152811.38	61805463.79	31966630.83
Phenyl acetate	C8H8O2	134.8935	852.5	1.5175817	neg	58188857.	24979923.5	41725060.1

				51		58	9	8
Nornicotine	C9H12N2	146.9638	767.3	6.68678167	neg	51960708.72	7925015.913	56132402.61
5-Aminopentanal	C5H11NO	102.0923	771.2	7.244779773	pos	71805698.53	12520638.09	31688487.04
Aminocarb	C11H16N2O2	209.1288	237	1.54928446	pos	38684574.87	37393357.77	37067719.17
Isovaleric acid	C5H10O2	102.0917	923.3	0.05213221	pos	36997597.03	38025369.32	36650390.9
Oxoadipic acid	C6H8O5	158.9781	177.4	5.93321748	neg	36697811.81	41296098.31	31762255
D-Xylitol	C5H12O5	152.0715	185.4	19.72756236	pos	38410872.79	36218735.86	34843784.04
trans-Cinnamate	C9H8O2	146.9651	610	0.12525568	neg	35599990	32784557.28	39304517.83
Perillyl alcohol	C10H16O	135.1179	364.2	7.27814767	pos	34836649.18	34458195.05	35057753.85
L-Valine	C5H11NO2	118.0864	295.8	1.05007857	pos	24904557.21	40955625.04	38377462
Histamine	C5H9N3	112.0868	202	0.67804593	pos	32316111.31	33445202.88	36360297.67
Caffeic acid	C9H8O4	181.0496	295.8	0.35901769	pos	34270791.91	32647022.09	34585488.97
3-Aminopentanedioate	C5H9NO4	128.0326	878.8	17.26093084	neg	42758555.05	35299197.16	21741641.69
Deoxycytidine	C9H13N3O4	228.0981	140.1	0.30345172	pos	32743804.68	33764062.44	31263239.99
(S)-3-Methyl-2-oxopentanoic acid	C6H10O3	131.0703	258.6	0.183107877	pos	30175232.2	30509629.09	29476237.63
Pyrroline	C5H7NO3	130.0496	842.6	2.122	pos	2988	50072	69665

hydroxycarboxylic acid				267197		3318.54	766.94	68.293
4-Hydroxycinnamoylmethane	C10H10O2	162.0768	161.2	3.818712431	pos	29176468.5	26427600.55	28673132.67
Abscisic aldehyde	C15H20O3	231.1384	439.4	20.04859426	pos	27960646.32	27754391.09	28544056.61
myo-Inositol	C6H12O6	179.0544	132.4	9.494321279	neg	15469642.31	16981562.04	51732698.53
Homovanillic acid	C9H10O4	182.9859	703	4.681788242	pos	36106076.8	39419525.05	8431777.185
13(S)-HpOTrE	C18H30O4	311.2219	595.5	0.076563137	pos	31852522.92	23960325.15	27932803.09
Stearic acid	C18H36O2	265.1457	883	8.485828312	neg	32279143.8	32478535.73	16446385.59
Fructose-1P	C6H12O6	179.0531	270.6	16.88884471	neg	39524224.71	26506799.94	14228958.76
Gabapentin	C9H17NO2	172.1335	611.7	0.083680963	pos	28096797.53	9217723.14	42793412.81
Methyl beta-D-galactoside	C7H14O6	176.9723	68.2	2.683912883	pos	22306038.49	22725150.67	33390025.3
2-trans,6-trans-Farnesal	C15H24O	221.1902	715.1	0.904199191	pos	27704053.15	25224781.7	24513047.81
Gulonic acid	C6H12O7	177.0378	806.6	11.91835868	neg	33263725.08	22760278.96	21351727.94
Aminoadipic acid	C6H11NO4	162.0768	130.5	3.445747238	pos	26564450.4	24466810.5	25202222.62
L-2,4-diaminobutyric acid	C4H10N2O2	118.0653	419	0.006252514	pos	27054815.94	24237632.44	24143867.59
(-)-beta-Pinene	C10H16	137.1325	596.9	0	pos	19103659.74	37479992.66	18692036.18

N(6)-Methyllysine	C7H16N2O2	161.1319	678.6	3.302803312	pos	25641537.5	24052108.55	25255937.43
(2Z,4S,5R)-2-Amino-4,5,6-trihydroxyhex-2-enoate	C6H11NO5	160.0608	138	28.32673584	pos	24313427.46	24315905.84	24240110.51
Tyrosol	C8H10O2	121.0648	252.4	1.987269454	pos	22045768.79	25534335.14	21416502.03
L-Rhamnono-1,4-lactone	C6H10O5	163.0603	160.8	1.373726822	pos	23851984.3	20961548.63	21406360.38
5-Acetamidovalerate	C7H13NO3	159.0804	610.4	26.41655387	pos	20719596.53	20233237.94	22550649.08
2-Methylserine	C4H9NO3	119.0496	228	0.010971494	pos	22715320.1	20137828.81	20382059.1
6-Methylmercaptopurine	C6H6N4S	166.0446	108.4	17.60670648	pos	20380424.45	20239613.02	21892994.88
3-Dehydroshikimate	C7H8O5	171.9933	344.9	0.037032402	pos	27344486.96	8886590.463	25941979.95
Phenylacetic acid	C8H8O2	135.9434	834.8	1.93331432	neg	24353301.9	24036888.11	13026540.93
Undecanoic acid	C11H22O2	186.9576	160.8	6.113738912	pos	14024779.1	24920262.78	21855570.5
N-Formyl-L-methionine	C6H11NO3S	176.972	610.3	0.00068977	pos	21400411.79	22637439.46	15879506.11
Stigmasterol	C29H48O	395.3693	848.3	3.793921278	pos	16626609.05	35188974.1	6774008.339
N-Acetyl-L-phenylalanine	C11H13NO3	208.0974	283.5	2.291208227	pos	19237566.65	18883439.53	18488883.83
12-Hydroxydodecanoic acid	C12H24O3	216.1961	579.5	3.278902478	pos	17809768.54	19219855.64	18940408.46
Thymidine	C10H14N2O5	243.0976	275.8	0.098	pos	1800	16701	17779

				725779		4670.85	016.58	180.02
Urocanic acid	C6H6N2O2	139.0509	194.5	1.820860901	pos	16225684.76	18981674.76	16797841.03
Jasmonic acid	C12H18O3	193.1229	629.2	24.51288791	pos	17197999.23	16042717.55	18461355.35
Dehydrocostus lactone	C15H18O2	231.1384	759.6	1.920927029	pos	16308303.11	17407848.11	17786792.72
L-Leucine	C6H13NO2	130.0864	531.2	1.092085183	neg	16109156.96	21509164.87	12601065.14
Gamma-terpinene	C10H16	137.1328	986.6	2.187660429	pos	19917852.38	24237930.94	4390490.032
Trioxsalen	C14H12O3	228.0846	179.6	17.28324059	pos	17530623.23	15332277.22	15445271.76
4-Guanidinobutanal	C5H11N3O	130.0861	984.4	1.311839159	pos	14576163.06	15403507.75	17709196.54
Pelargonic acid	C9H18O2	158.1545	178.2	5.818104088	pos	18330083.79	16787160.92	12113493.92
5-Amino-2-oxopentanoic acid	C5H9NO3	131.0702	209.8	1.145391828	pos	14731109.96	14780681.31	17291874.92
Neocnidilide	C12H18O2	195.1384	623.6	0.875330155	pos	15350957.76	14710806.6	14831489.79
7-Methylguanine	C6H7N5O	166.0725	178.2	0.005550642	pos	15979986.68	14654665.1	14220685.4
o-Toluate	C8H8O2	135.0428	576.6	17.20935881	neg	15563956.92	23509668.01	5299708.364
Dodecanedioic acid	C12H22O4	213.149	596.4	12.75021126	pos	14406685.82	13303293.23	16610386.96
3,4-Methylenedioxyamphetamine	C11H15NO2	194.1179	261.6	0.06332482	pos	14661086.36	13943057.1	14925451.69

5'-Methylthioadenosine	C11H15N5O3S	297.2425	845.3	2.952759269	neg	12861775.7	26604041.01	3925818.085
Azelaic acid	C9H16O4	171.1495	967.1	3.087606934	pos	6611859.676	28181924.5	7451970.345
2-Keto-6-aminocaproate	C6H11NO3	145.0764	377.7	17.23229967	pos	17317161.74	12822035.97	12083546.6
Methyl jasmonate	C13H20O3	225.1488	496.5	0.207228415	pos	14831487.69	13206822.37	14150891.9
Linatine	C10H17N3O5	259.13	199	4.689061027	pos	15287991.62	13612727.02	13118602.55
Phloretin	C15H14O5	273.0771	877.9	1.01070357	neg	13879914.45	21634219.24	6056030.45
Ubiquinone-1	C14H18O4	233.1176	458.1	19.41052928	pos	14286411.5	12481629.89	13818161.29
N-Acetylornithine	C7H14N2O3	175.1122	550.9	22.46462212	pos	12021047.87	10774880.6	16480400.85
Gingerol	C17H26O4	295.1885	439.1	6.35526113	pos	15525680.63	7295976.322	16292489.24
Spermidine	C7H19N3	145.1014	576.6	27.09430025	pos	16655805	4044227.892	16146677.33
Adenine	C5H5N5	134.0457	388.5	2.683304127	neg	12352191.57	11655015.12	11613881.13
Protocatechuic acid	C7H6O4	153.0189	710.9	2.770899543	neg	6692977.106	20613756.62	7862294.14
L-Fucose	C6H12O5	163.059	98.2	7.585183461	neg	12934252.7	10222401.09	11842584.36
2-Hydroxyphenylacetate	C8H8O3	151.0385	390.7	10.09014258	neg	15618060.95	15922495.24	1959516.542
Tropate	C9H10O3	167.0705	763.8	1.3407513	pos	12313671.	10435164.6	10437953.7

				59		39	6	8
4-Hydroxyphenylpyruvic acid	$C_9H_8O_4$	180.0438	592.3	2.752543842	pos	10917612	10188697.93	12061369.52
2-Dehydropantoate	$C_6H_{10}O_4$	146.0604	286.6	0.033808164	pos	10741169.44	11695963.3	10603591.94
Citraconic acid	$C_5H_6O_4$	129.0176	386.8	13.17649685	neg	9326164.607	8520943.037	14856081.54
Chavicol	$C_9H_{10}O$	135.0808	629.2	0.054855679	pos	9066847.468	11612894.54	11813103.5
11-Dehydrocorticosterone	$C_{21}H_{28}O_4$	325.1735	878.7	21.25019413	neg	6543114.468	10122364.04	15805641.69
3-Hydroxyanthranilic acid	$C_7H_7NO_3$	152.034	106.1	1.522389347	neg	10733741.16	10630568.83	10838200.56
D-Alanyl-D-serine	$C_6H_{12}N_2O_4$	176.0802	119.3	2.83961513	pos	11263178.85	11317985.1	7998682.856
S-Adenosylmethionine	$C_{15}H_{22}N_6O_5S$	398.2451	733.3	7.987616834	pos	21717210.84	4410917.327	4210760.268
9(S)-HOT	$C_{18}H_{30}O_3$	277.2168	623.6	2.317351369	pos	9628353.643	9465557.114	10664021.93
gamma-Glutamyl-beta-aminopropionitrile	$C_8H_{13}N_3O_3$	200.0923	264.9	3.048275836	pos	3756206.058	4316966.146	21096836.32
D-Proline	$C_5H_9NO_2$	114.0533	661.5	23.88357023	neg	13400400.27	5914846.962	9612049.459
Xanthoxic acid	$C_{15}H_{22}O_4$	266.1472	559.2	17.28366859	pos	9045002.782	6552872.793	12900154.47
Aspartame	$C_{14}H_{18}N_2O_5$	295.1296	351.6	0.018035585	pos	10682485.82	8968735.923	8485707.193
(+)-(S)-Carvone	$C_{10}H_{14}O$	151.1115	32.7	1.323526006	pos	8740775.76	9816185.835	9069863.6

3-Hydroxybenzoic acid	C7H6O3	137.0225	194.5	13.99808061	neg	10597109.53	13544146.6	3482648.029
Oleic acid	C18H34O2	281.2476	834.9	3.640919958	neg	7258459.831	10014374.51	10062928.22
Perindopril	C19H32N2O5	367.231	786.5	19.54083397	neg	11588184.26	9387589.784	6276855.747
D-Fructose	C6H12O6	180.1025	202	4.793076559	pos	9237966.521	9146945.863	8401156
Theophylline	C7H8N4O2	180.0657	366.7	5.553528518	pos	8965714.452	8520944.831	8846242.356
N-Acetylglutamic acid	C7H11NO5	190.0715	208.3	2.630589015	pos	9158349.51	8099971.864	8614348.377
Erythritol	C4H10O4	121.0266	218.2	14.42327809	neg	8876489.444	14288571.98	2683714.183
N2-Succinyl-L-arginine	C10H18N4O5	275.135	160.8	0.087229905	pos	8949077.188	8465993.678	8367450.594
Pyrrole-2-carboxylic acid	C5H5NO2	111.5415	689.9	6.531341474	pos	7839974.827	9349456.14	8237728.427
Norsanguinarine	C20H15NO4	333.115	249.4	1.49903835	pos	8092902.095	9068369.422	7876535.172
Procollagen 5-hydroxy-L-lysine	C7H13N3O3R2	197.8067	665.7	6.796704025	neg	2816525.448	14530217.94	7519621.126
Ketoleucine	C6H10O3	130.0083	964.8	2.693450326	pos	5656260.436	6719329.542	12377199.92
Diglycidyl resorcinol ether	C12H14O4	222.0835	379.8	25.66602201	pos	10796510.66	10938300.6	2800030.447
Arachidic acid	C20H40O2	311.1673	786.8	3.522208452	neg	10578985.76	10769831.21	2618923.251
Illudin M	C15H20O3	248.1363	552.9	19.747211	pos	7527765.3	7244507.11	8632046.27

				51		31	2	1
L-Kynurenine	C10H12N2O3	208.1339	305.1	2.647983241	pos	8285616.719	7059777.957	7234951.023
Gluconic acid	C6H12O7	197.154	784.8	0.634656649	pos	6935108.148	6878754.62	8159280.557
Gluconolactone	C6H10O6	177.0385	468.6	10.86769262	neg	2050145.1	8833528.705	10727045.17
D-Mannonate	C6H12O7	196.0609	407.9	13.26136154	pos	6713259.37	6359456.026	7309921.764
Metoclopramide	C14H22ClN3O2	300.148	530.6	2.078974373	pos	5247475.853	9379244.584	5179042.254
Mesaconate	C5H6O4	129.0176	667.6	13.36251798	neg	11330704.6	3783010.827	4318922.557
Benzoate	C7H6O2	121.0274	456.6	17.54974493	neg	1935175.746	8342879.865	9022960.769
Shikimic acid	C7H10O5	175.0284	208.2	5.104521958	pos	2011496.633	7989635.094	8276206.601
Cellobiose	C12H22O11	342.1385	225	1.357893186	pos	7249658.86	4684630.109	6108438.502
Phenylethylamine	C8H11N	121.0266	239.5	12.19708622	neg	8701890.112	7302595.32	1869420.044
Methylmalonic acid	C4H6O4	117.017	824.4	19.86036217	neg	10284306.13	4818450.58	2075045.734
2-Oxo-4-phenylbutyric acid	C10H10O3	179.0707	514.9	2.367779877	pos	5690261.748	5359198.456	6119311.368
Prephenate	C10H10O6	226.0714	191.5	12.35722108	pos	5813654.784	4721657.743	4488183.462
3,4-Dihydroxyphthalate	C8H6O6	197.0074	118.9	8.750863742	neg	5859870.097	4865155.656	4200925.692
Alpha-D-Glu	C6H12O6	161.0432	716.8	11.23	ne	1303	70835	64589

cose				922028	g	615.095	25.873	49.223
(-)-5'-Demethylyatein	C21H22O7	387.1437	505.8	0.010174227	pos	5285903.333	4186574.775	5145259.11
6-Hydroxynicotinic acid	C6H5NO3	138.0178	716	13.21568667	neg	3045969.904	4703593.058	6831241.554
3-Hydroxyphenylacetic acid	C8H8O3	151.0386	316.9	9.428053491	neg	5874025.232	3663473.352	4196083.13
16-Oxopalmitate	C16H30O3	253.2168	793	18.69544201	pos	5524034.829	1682124.229	6178419.231
8,9-EET	C20H32O3	303.2321	569.5	14.62246246	pos	5123445.21	3650426.798	4385545.023
Creatinine	C4H7N3O	112.9837	686.4	5.434101061	neg	9407135.822	1364384.231	2283703.542
Itaconic acid	C5H6O4	129.0177	176.6	12.40139919	neg	4118612.139	4608163.65	4276912.2
Cytisine	C11H14N2O	191.1182	246.2	1.242004508	pos	5512814.471	5208041.867	1851698.93
Sphinganine	C18H39NO2	284.2948	871.9	1.648731996	pos	6947281.969	4653519.65	887367.3745
Citrulline	C6H13N3O3	176.1073	311.4	21.24858714	pos	4738714.979	3523855.098	3482089.506
Citramalic acid	C5H8O5	147.0286	237.3	8.823834274	neg	1258631.611	9090709.779	1308060.564
Kaempferol	C15H10O6	287.055	614.5	0.083607671	pos	4324092.852	3072722.66	3110338.553
p-Anisic acid	C8H8O3	151.0385	425.6	10.09014258	neg	3452520.96	3100308.87	3621139.611
Uric acid	C5H4N4O3	167.0192	105.8	5.535011585	neg	4475329.713	3556731.484	1478505.651

Linoleic acid	C18H32O2	279.2321	900.8	2.950950124	neg	3279905.308	2776634.302	2949887.378
1-Naphthol	C10H8O	145.0651	418.2	2.233480003	pos	1630730.46	6010363.474	1354509.744
N-Acetylleucine	C8H15NO3	172.0973	287.1	3.625855839	neg	2774780.817	2287390.767	2701070.682
L-2-Hydroxyglutaric acid	C5H8O5	147.0277	744.7	15.12640135	neg	3123345.343	2285781.599	2216037.341
5-Methyl-2'-deoxycytidine	C10H15N3O4	240.0979	253.7	4.681423702	neg	2691889.291	2353970.851	2565317.227
D-Gulono-1,4-lactone	C6H10O6	178.0487	314.7	3.931508627	neg	2707500.204	2298619.008	2254321.874
D-Arabitol	C5H12O5	151.0595	659.5	4.694981302	neg	627675.8724	3157235.828	3397579.244
Pyridoxine	C8H11NO3	170.0933	234	10.36052484	pos	2905134.913	1462975.735	2441402.135
Isoeugenol	C10H12O2	163.0749	440.7	9.345398955	neg	2412964.667	1774207.883	2324510.834
Phenylpyruvic acid	C9H8O3	164.0437	234.5	7.53250215	neg	2082368.698	1898050.449	2102741.64
Ascorbate	C6H8O6	159.0291	229.4	27.88168958	pos	2491986.141	2021531.48	1526607.416
Biochanin A	C16H12O5	283.0596	377.7	5.643631235	neg	2005000.251	1908665.683	1852309.383
Alternariol	C14H10O5	257.0441	339.8	5.446536217	neg	1948194.531	1614136.368	1741756.601
4-Methylumbelliferone	C10H8O3	175.0387	927.9	7.564041552	neg	299453.9859	230902.6531	3868241.32
Suberic acid	C8H14O4	173.0805	929.2	0.0126973	neg	375319.58	2081070.34	1092029.10

				63		35	5	8
Glycyl-glycine	C4H8N2O3	131.0446	293.6	2.842984663	neg	280935.3059	214776.924	237666.4726

*: m/z: mass-to-charge ratio; RT: retention time; ppm: error between the detected molecular weight and the theoretical molecular weight in ppm; pos/neg: positive/negative; X (mean ± SD): mean ± standard deviation of the distribution of signals of the metabolite in a given type.

References

S1. Zelena, E., Dunn, W.B., Broadhurst, D., Francis-McIntyre, S., Carroll, K.M., Begley, P., O'Hagan, S., Knowles, J. D., Halsall, A., Wilson, I. D., Kell, D. B. Development of a Robust and Repeatable UPLC−MS Method for the Long-Term Metabolomic Study of Human Serum. *Analytical Chemistry* **81**, 1357-1364(2009).

S2. Want, E. J., Masson, P., Michopoulos, F., Wilson, I. D., Theodoridis, G., Plumb, R. S., Shockcor, J., Loftus, N., Holmes, E., Nicholson, J.K. Global Metabolic Profiling of Animal and Human Tissues via UPLC-MS. N*ature Protocols* **8**, 17-32(2013).
